# Consistent, small effects of treefall disturbances on the composition and diversity of four Amazonian forests

**DOI:** 10.1111/1365-2745.12529

**Published:** 2016-01-17

**Authors:** Timothy R. Baker, Dilys M. Vela Díaz, Victor Chama Moscoso, Gilberto Navarro, Abel Monteagudo, Ruy Pinto, Katia Cangani, Nikolaos M. Fyllas, Gabriela Lopez Gonzalez, William F. Laurance, Simon L. Lewis, Jonathan Lloyd, Hans ter Steege, John W. Terborgh, Oliver L. Phillips

**Affiliations:** ^1^ School of GeographyUniversity of LeedsWoodhouse LaneLeedsLS2 9JTUK; ^2^ Department of BiologyWashington University in St. LouisSaint LouisMO63130USA; ^3^ Universidad San Antonio Abad del CuscoAvenida de la CulturaCuscoPeru; ^4^ Universidad Nacional de la Amazonía PeruanaSargento Lores 385IquitosPeru; ^5^ Prolongación Bolognesi Mz‐E‐6OxapampaPasco, Peru; ^6^ School of Marine and Tropical BiologyJames Cook UniversityCairnsQld4870Australia; ^7^ Department of Ecology and SystematicsFaculty of BiologyUniversity of AthensPanepistimiopolis15701AthensGreece; ^8^ Biological Dynamics of Forest Fragments ProjectNational Institute for Amazonian Research (INPA) and Smithsonian Tropical Research InstituteAv. André Araujo2936ManausBrazil; ^9^ University College LondonGower StreetLondonWC1E 6BTUK; ^10^ Imperial CollegeSilwood Park Campus, Buckhurst RoadAscotWest BerkshireSL5 7PYUK; ^11^ Naturalis Biodiversity CenterPO Box 95172300 RALeidenThe Netherlands; ^12^ Center for Tropical ConservationNicholas School of the EnvironmentDuke UniversityBox 90328DurhamNC27708USA

**Keywords:** alpha‐diversity, beta‐diversity, determinants of plant community diversity and structure, functional composition, maximum height, seed mass, tropical forest, wood density

## Abstract

1. Understanding the resilience of moist tropical forests to treefall disturbance events is important for understanding the mechanisms that underlie species coexistence and for predicting the future composition of these ecosystems. Here, we test whether variation in the functional composition of Amazonian forests determines their resilience to disturbance.
2. We studied the legacy of natural treefall disturbance events in four forests across Amazonia that differ substantially in functional composition. We compared the composition and diversity of all free‐standing woody stems 2–10 cm diameter in previously disturbed and undisturbed 20 × 20 m subplots within 55, one‐hectare, long‐term forest inventory plots.
3. Overall, stem number increased following disturbance, and species and functional composition shifted to favour light‐wooded, small‐seeded taxa. Alpha‐diversity increased, but beta‐diversity was unaffected by disturbance, in all four forests.
4. Changes in response to disturbance in both functional composition and alpha‐diversity were, however, small (2 – 4% depending on the parameter) and similar among forests.
5. *Synthesis*. This study demonstrates that variation in the functional composition of Amazonian forests does not lead to large differences in the response of these forests to treefall disturbances, and overall, these events have a minor role in maintaining the diversity of these ecosystems.

## Introduction

Disturbance events have an important role in explaining species coexistence and spatial patterns of composition and diversity in plant communities (Eggeling 1947; Grime 1973; Connell 1978; Wilkinson 1999; Shea, Roxburgh & Rauschert 2004). Undoubtedly, the most influential concept underlying this work has been the intermediate disturbance hypothesis (Grime 1973; Connell 1978; Sheil & Burslem 2003, 2013; Fox 2013). According to this hypothesis, as the frequency, size, or intensity of disturbance decreases, diversity rises and then falls as community composition changes from being characterized by a high abundance of species with greater colonizing ability to a greater abundance of species that are better competitors (Grime 1973; Connell 1978). This hypothesis was proposed, in part, to explain one of the central issues in ecology – the great diversity within and among moist tropical forests – and the individual and multiple treefalls that regularly occur in these ecosystems were a testing ground for the development of these ideas (e.g. Connell 1978).

Since the hypothesis was first outlined, disturbance has been shown to have a range of impacts on the composition and diversity of moist tropical forests. For example, some studies have demonstrated a positive (Schnitzer & Carson 2001) or hump‐backed (Molino & Sabatier 2001) relationship between forest diversity and disturbance. However, other studies have found that disturbance has little (Bongers *et al*. 2009) or no (Hubbell *et al*. 1999) effect on community composition or overall diversity. This variability in the impact of disturbance within moist tropical forests reflects findings from other ecosystems: a large number of studies now indicate that hump‐backed diversity/disturbance relationships are only found in a minority of cases (e.g. approximately 20%, Hughes *et al*. 2007) and that positive, negative, U‐shaped or, most commonly, no relationship may also be found (Hughes *et al*. 2007). A key current question is therefore why the effect of disturbance on composition and diversity varies so much (Mackey & Currie 2001; Sheil & Burslem 2003; Shea, Roxburgh & Rauschert 2004; Hughes *et al*. 2007).

One possible answer to this question is that the traits of the species present in any community determine how disturbance affects diversity and composition (Shea, Roxburgh & Rauschert 2004; Cadotte 2007; Haddad *et al*. 2008). This hypothesis is based on the well‐known relationships between the life‐history traits of individual species and response of those species to disturbance (Grime 1973; Moloney & Levin 1996). For example, communities dominated by slow‐growing species with poor colonization abilities may be affected more strongly by disturbance than communities dominated by fast‐growing species with greater dispersal ability. Developing and testing such a framework for predicting the effect of treefall disturbances is particularly important for tropical forests, as increasing rates of disturbance characterize all of the major threats to these ecosystems, whether ultimately related to logging (Asner *et al*. 2009), drought (Phillips *et al*. 2009), fire (Brando *et al*. 2014) or fragmentation (Laurance *et al*. 1998). If functional composition is a useful predictor of how intact tropical forests respond to disturbance, it would be a powerful basis to predict the resilience of different communities to these threats. However, to date, there has been no test of this hypothesis in the field across different sites.

In this study, we therefore compared the effect of treefall disturbance events on forest composition and diversity in four contrasting sites that sample the full range of functional composition that occurs within upland forests in Amazonia. Rather than a test of the intermediate disturbance hypothesis, this study therefore explores how functional composition influences the resilience of these communities to disturbance. There is wide variation in the functional composition of Amazonian forests (e.g. Baker *et al*. 2004; ter Steege *et al*. 2006) and highly variable and sometimes exceptional alpha (Gentry 1988; ter Steege *et al*. 2003) and beta‐diversity (Tuomisto *et al*. 1995). These patterns make Amazonia a valuable and important location for testing this idea.

The variation among Amazonian forests in the abundance of species with different growth rates and traits related to colonization ability is particularly relevant to this study. For example, western Amazon forests have lower community‐level wood density and contain more small‐statured tree species compared to forests in central and eastern Amazonia (Baker *et al*. 2004, 2009; Quesada *et al*. 2012). In contrast to both of these regions, forests on the Guiana Shield have high community‐level wood density and taller statured tree species with high seed mass (ter Steege *et al*. 2006; Feldpausch *et al*. 2011). The origin of these differences reflects both the contrasting evolutionary history and current ecology of the different regions of Amazonia. For example, the functional composition of western Amazon forests is associated with comparatively young, rich soils derived from the Andean uplift and deposition during the Miocene (Hoorn *et al*. 2010; Higgins *et al*. 2011), whereas the composition of forests on the Guiana Shield is dominated by a specific group of tall‐statured caesalpinioid legume species with dense wood (ter Steege *et al*. 2006). Overall, these patterns mean that forests in central Amazonia and the Guiana Shield have a higher proportion of slow‐growing tree species, compared to western Amazon forests (Fyllas, Quesada & Lloyd 2012). Based on these geographical patterns of functional composition, we therefore predicted that the greatest change in composition and diversity following disturbance events would be found in the sites in central Amazonia and the Guiana Shield. In western Amazonia, we expected that treefall disturbances would have little effect on the forest's functional composition, or, as a result, its diversity.

## Materials and methods

### Field Sampling Strategy

We compared the effect of treefall disturbance events within permanent forest plots among four sites across Amazonia: two sites in the western Amazon (north and south Peru), one site in central Amazonia (Manaus) and one site in French Guiana (Nouragues; Tables 1 and S1 in Supporting Information). We compared forest structure, composition and diversity between subplots where tree mortality events had opened a canopy gap 5–10 years prior to this study, creating both gap and shaded habitats within a single patch of forest, with subplots that had remained undisturbed. Our sampling strategy therefore examines whether the largest disturbance events that occur regularly (≈ every five years within a single hectare of forest) significantly augment diversity and alter forest composition at the four sites. Treefall events of this scale dominate tree mortality in Amazonia (Espírito‐Santo *et al*. 2014).

**Table 1:** Characteristics of the study sites. Plot locations are the areas where inventory plots are located (for more details of individual plots, see Table S1). Date of fieldwork are the mean ± 95% CI for the start date of the inventory for each site; time since disturbance the mean time elapsed between this date and the mid‐year of the census interval when high tree mortality occurred in the disturbed subplot. For stem number, wood density and species richness, mean ± 95% CI of values for control subplots are shown

Site	Country	Region	Plot locations	Date of fieldwork	Time since disturbance (years)	Total pairs	Control subplots (20 × 20 m)		
							No. stems 2–10 cm	Wood density (g cm^−3^)	Species richness
S Peru	Peru	West	Explorers Inn						
			Inkaterra	2007.15 ± 0.36	5.74 ± 1.1	15	86 ± 8	0.595 ± 0.014	45 ± 5
			Cocha Cashu						
N Peru	Peru	West	Allpahuayo						
			Sucusari	2006.97 ± 0.07	5.91 ± 1.6	11	107 ± 19	0.611 ± 0.017	62 ± 9
			Yanamono						
Manaus	Brazil	East	BDFFP	2007.54 ± 0.02	6.53 ± 0.4	13	112 ± 11	0.671 ± 0.013	70 ± 6
Nouragues	French Guiana	East	Nouragues	2007.98 ± 0.20	9.76 ± 1.6	10	71 ± 15	0.698 ± 0.024	46 ± 8

Our sampling used permanent forest plots that form part of the RAINFOR network (Malhi *et al*. 2002) which provides a unique historical record of forest dynamics at these sites. Using long‐term inventory data for trees ≥10 cm diameter for each site, we identified 20 × 20 m patches of forest within one‐hectare plots that (i) had experienced a substantial tree mortality event (disturbed subplots) and (ii) had not been affected by tree mortality since the beginning of monitoring, 16.6 ± 1.6 years ago across all sites (control subplots).

Subplot selection proceeded in three steps:

1. Prior to visiting each plot, the annual rate of basal area loss was calculated for each census interval for each subplot. These data were compiled to create the distribution of annual rates of basal area loss at a scale of 20 × 20 m for each plot. As mortality events of large trees are rare, these distributions are highly skewed, with few census intervals and subplots experiencing high rates of basal area loss. In order to sample these rare events, we therefore chose subplots that had a single census interval in the upper 10% of this distribution for each plot as potential ‘disturbed’ subplots; subplots with consistently low rates of basal area loss (outside the upper 10% of this distribution throughout monitoring) were considered as potential ‘control’ subplots. Subplots that had high rates of basal area loss in more than one census interval were excluded from the study.
2. In the field, these classifications were validated. Firstly, all subplots were divided into 10 × 10 m quadrats, and each quadrat scored as ‘mature’, ‘intermediate’ or ‘gap’ phase forest (following Whitmore 1998). Disturbed subplots were successfully validated if they contained at least two 10 × 10 m quadrats classified as ‘intermediate’ or ‘gap’ phase forest. Control subplots were successfully validated if they had zero or only one ‘intermediate’ or ‘gap’ quadrat. This protocol ensured that the original gap size in the disturbed subplots was consistent across all sites (approximately 200–400 m^2^ at the canopy level).
3. Finally, the validated subplots were examined in the field to confirm (i) for disturbed subplots, the presence of trunks that matched the number, size and/or species identity of mortality events recorded in the inventory data and (ii) for both disturbed and control subplots, the lack of any major, recent mortality events that had not been recorded since the most recent plot census.

All validations were performed independently by TRB and one other person, and both had to agree on the classification of a subplot as ‘disturbed’ or ‘control’ for it to be considered for sampling. Overall, 55 plots were examined, covering a total of 72.2 hectares. Following validation, some of the one‐hectare plots contained no suitable ‘disturbed’ and ‘control’ subplots for this study, and these plots were excluded. Where plots contained multiple pairs of successfully validated ‘control’ and ‘disturbed’ 20 × 20 m subplots, a pair was selected at random. In Nouragues and Manaus, some of the one‐hectare plots are contiguous; in some cases, we therefore paired nearby disturbed and control subplots from adjacent plots. In total, 49 disturbed/control subplot pairs were selected (15 S Peru, 11 N Peru, 13 Manaus, 10 Nouragues).

The average time since the major treefall event(s) in the disturbed subplots until our assessment of forest composition and diversity was 6.8 ± 0.7 years, calculated to the mid‐year of the census interval when the tree mortality occurred. During the period of high mortality in the disturbed subplots, the annual rate of basal area loss was 11.1 ± 2.0% (Fig. S1). In the context of these subplots, such mortality rates are extreme – the death of some of the very largest trees in the forest – and represent a reduction in the total basal area of these subplots by 0.63 ± 0.13 m^2^, which is equivalent to the death of one tree ≈ 90 cm diameter in a 20 × 20 m patch of forest. In contrast, prior to this census interval, the rate of basal loss was low and similar in both disturbed and control subplots (Fig. S1). During more recent census intervals, tree mortality remained marginally higher in the disturbed compared to the control subplots, possibly because trees damaged during the previous major mortality event also died (Fig. S1).

### Floristic Data

We focussed our study on the effect of disturbance on all woody stems 2–10 cm diameter – the ‘future forest’; other studies of disturbance/diversity relationships in tropical forests have focussed on the same (Molino & Sabatier 2001) or similar stem size classes (e.g. 1–3.9 cm, Hubbell *et al*. 1999). Although there may be strong impacts of disturbance on smaller seedling and saplings, the majority of these individuals die before reaching maturity. The timeframe and size classes used in this study were chosen to examine those individuals which had survived these early life‐history stages and, despite ongoing competition‐related mortality, were therefore more likely to reach reproductive maturity. If the effect of disturbance on the diversity of this cohort is small, then the legacy of treefall disturbance events for the diversity of larger trees is likely to be negligible. All stems 2–10 cm diameter were inventoried following standard procedures for small trees and lianas (Condit 1998; Gerwing *et al*. 2006). Botanical collections were made from unidentified stems and identified and stored in nearby herbaria. All morphospecies were consistently identified within different sites (i.e. N Peru, S Peru, Manaus, Nouragues). All free‐standing woody stems were included in the inventory, which included a few juvenile lianas that have a sapling growth‐form when young (e.g. *Salacia macrophylla*). The very small proportion of individuals (2.9%) that had multiple stems means that analyses on a stems or individual basis gave very similar results; all analyses presented here were conducted on a stems basis. In total, 10 180 stems are included in this study, including 1128 named species.

### Trait Data

For each subplot, community‐level mean values for wood density, seed mass, maximum height, intrinsic foliar nitrogen concentration and leaf mass per unit area (LMA) were calculated using published data. The selected traits are all related to species’ competitive and/or colonizing ability (e.g. wood density values are typically inversely related to high maximum growth rates; Muller‐Landau 2004; Poorter *et al*. 2008). Published values, rather than direct measurements for every stem, underestimate the variance of trait values within a plot but provide good estimates of community‐level means (Baraloto *et al*. 2010). We therefore used these data to compare the effect of disturbance on community‐level mean values, but did not explore the effects on functional diversity. We also acknowledge that the trait values that we used were measured for adult trees and may vary during ontogeny. However, the approach is consistent with our aim to assess how disturbance will influence the future functional composition of these forests.

Average community‐level trait values were calculated on a stems basis. Wood density data was sourced from a species‐level data base where wood density was measured as dry mass/fresh volume (Zanne *et al*. 2009). Seed mass was compiled from data from ter Steege *et al*. (2006); maximum height data were extracted from Baker *et al*. (2009) and values for intrinsic foliar nitrogen and LMA were used from Fyllas *et al*. (2009). Trait values were allocated to stems in the plot data based on matching species‐ or genus‐level values following Baker *et al*. (2004): 90.0, 85.3, 88.8, 75.2 and 75.8% of stems have species‐ or genus‐level for wood density, seed mass, maximum height, foliar nitrogen concentration or LMA, respectively. Remaining stems were allocated family‐level values or, where unidentified, were allocated the mean trait value of all stems with a species‐level value in the data set.

### Data Analysis

Firstly, we explored whether variation in the species and functional composition of the understorey tree communities matched the patterns for canopy trees that provided the basis of our sampling strategy. We compared the stem number, mean community‐level trait values and alpha‐diversity, using Fisher's alpha and the Shannon index, of the control subplots of the four sites. We used principal coordinates analysis with the Bray Curtis measure of floristic dissimilarity calculated using the species abundances for each site to explore variation in species composition in the understorey among the four forests.

We then investigated the effect of disturbance at each site by comparing the structure, composition and diversity of the disturbed compared to the control subplots. Firstly, paired *t*‐tests were used to examine whether there were significant differences in stem number, community‐level mean trait values and alpha‐diversity between control and disturbed subplots across all sites and within each forest. In addition to using Fisher's alpha and the Shannon index for these calculations, we also compared changes in diversity within sites using estimates of species richness that were rarefied to the minimum number of stems in a single subplot in each location. We standardized our species richness values to a different number of stems for each site because there were substantial differences in the density of individuals among the four forests. A Bonferroni‐corrected *P* value of 0.0125 was used to judge significance of the paired *t*‐tests for each parameter within each of the four sites.

Secondly, linear mixed‐effect models (Galway 2006) were used to test whether there were significant differences among sites (treated as random factors) in the changes in functional composition and alpha‐diversity, whilst controlling for the effect of the age and intensity of disturbance (treated as fixed factors). We used likelihood ratio tests to assess whether models that included site as a factor performed better than models that excluded this term. The intensity of disturbance in the disturbed subplots was quantified as the absolute loss in basal area during the census interval with high mortality (Fig. S1). The age of disturbance was calculated as the time since the mid‐year of the census interval with high mortality to the date of fieldwork for this study.

Finally, we tested whether the disturbed subplots had more variable species composition (greater beta‐diversity) than the control subplots in each site, by comparing the dispersion of disturbed and control subplots within a principal coordinates ordination of species composition (Anderson, Ellingsen & McArdle 2006).

All analyses were carried out using R (R Development Core Team 2012) and the *vegan* (Oksanen *et al*. 2015) and *lme4* packages (Bates *et al*. 2015).

## Results

**Figure 1:**
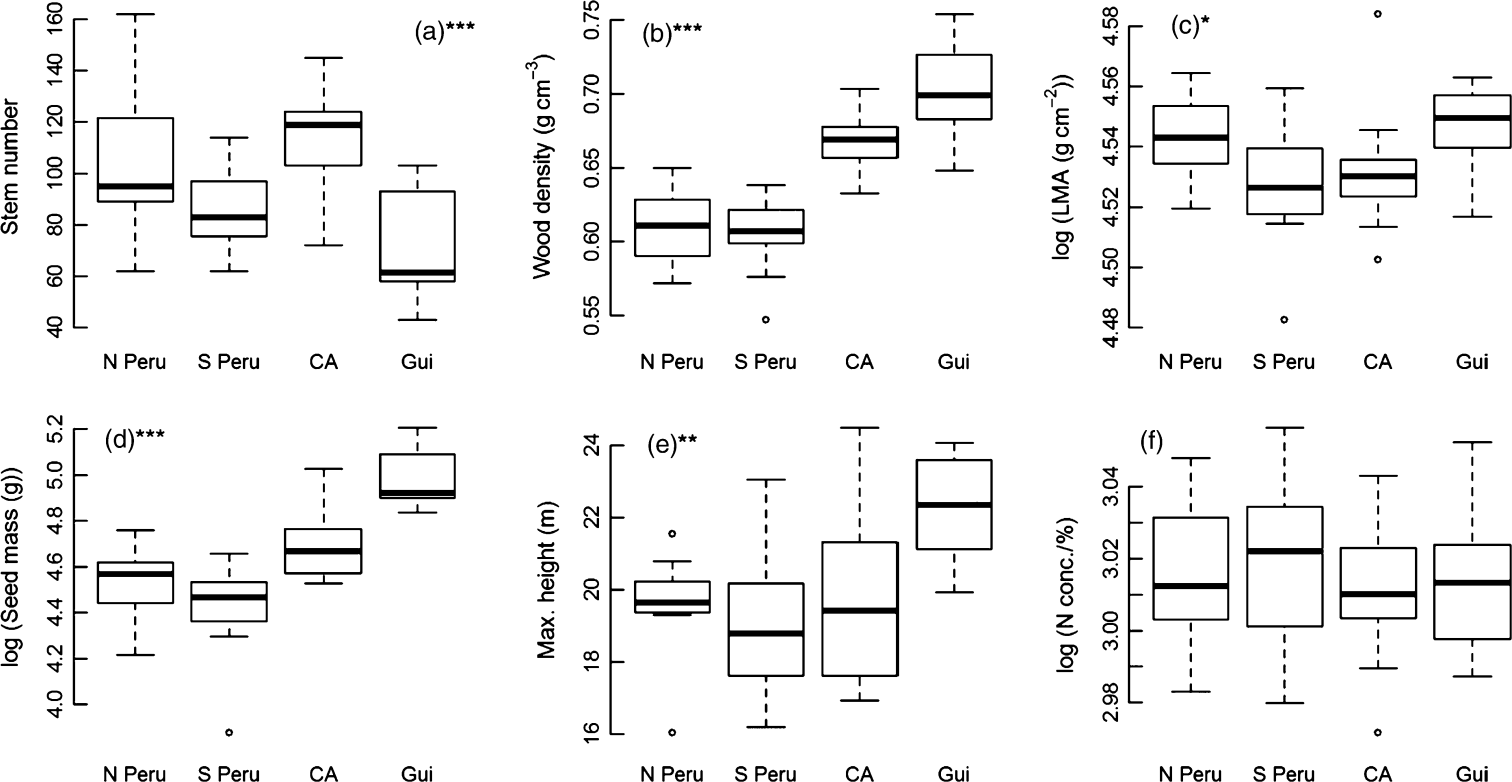
Box plots of variation in (a) stem density and mean values of (b) wood density, (c) leaf mass per unit area, (d) seed mass, (e) maximum height and (f) mean nitrogen concentration for stems 2–10 cm diameter in forests in four regions of Amazonia (north and south Peru, Central Amazonia (Manaus; CA) and the Guiana Shield (Nouragues; Gui). Significant differences among sites shown as *P *<* *0.05 (*), *P < *0.01 (**), *P *<* *0.001 (***).

There were significant differences in wood density, seed mass, maximum height and LMA among the understorey trees in the control subplots from each site (Fig. 1; wood density, *F*_3,45_ = 36.4, *P *<* *0.001; seed mass *F*_3,45_ = 23.7, *P *<* *0.001; maximum height, *F*_3,45_ = 6.4, *P *<* *0.005; LMA, *F*_3,45_ = 3.0, *P *<* *0.05). These differences largely mirrored the patterns found previously for trees ≥10 cm diameter and are principally driven by high values for these traits in Nouragues in French Guiana (Fig. 1). Understorey stem density also varied markedly among sites (Fig. 1; stems, *F*_3,45_ = 9.0, *P *<* *0.001). Stem density was lowest in the plots in Nouragues, which is probably related to the very high values of above‐ground biomass of trees ≥10 cm diameter in this region of Amazonia (Mitchard *et al*. 2014) and therefore greater shade in the understorey in this site.

**Figure 2:**
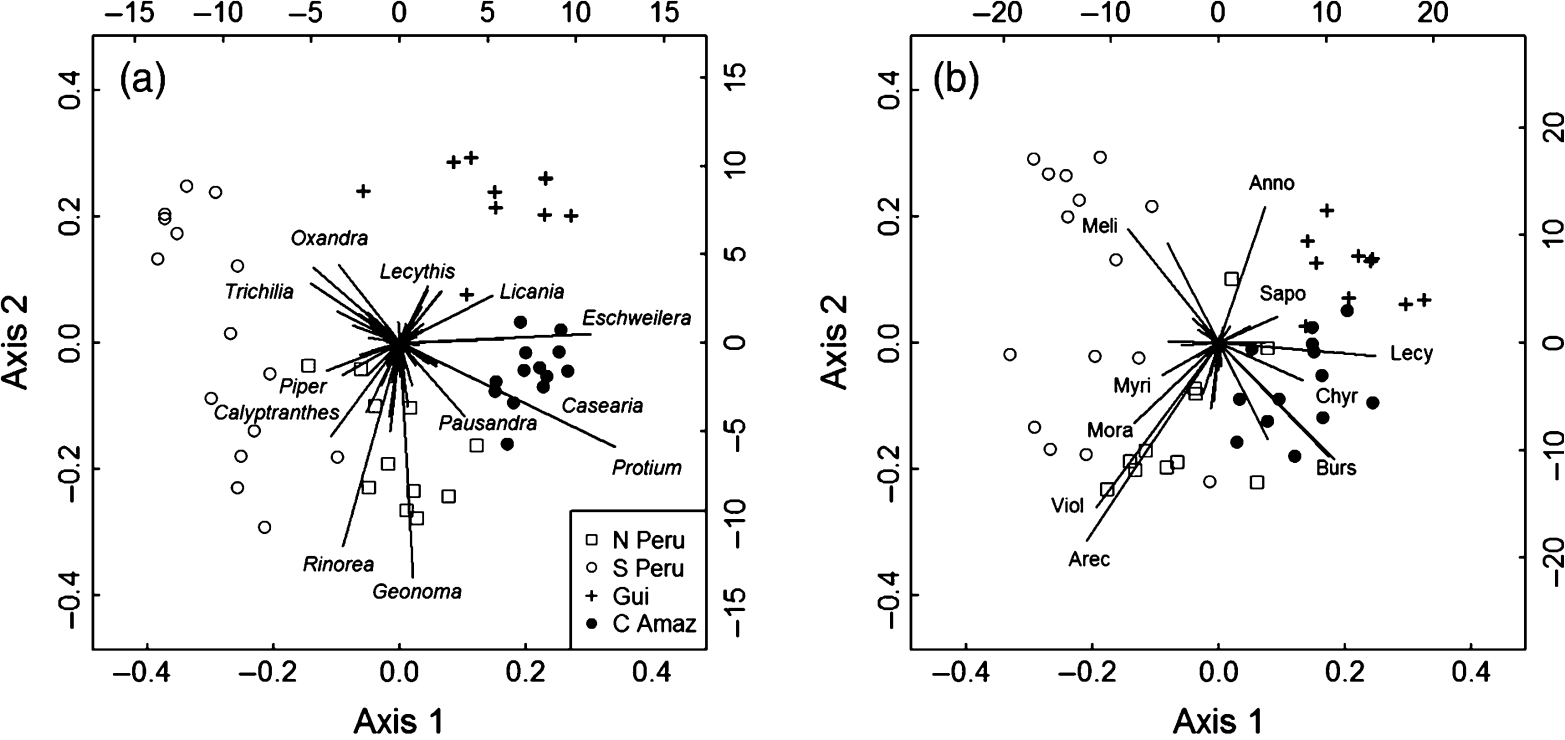
Principal coordinates analysis using Bray Curtis dissimilarity measure of the variation in (a) genus‐level and (b) family‐level composition of stems 2–10 cm diameter in undisturbed, control 20 × 20 m subplots of four Amazonian forests (north and south Peru, Central Amazonia (Manaus; CA) and the Guiana Shield (Nouragues; Gui). In each graph, the points represent sites, and the lines represent the ordination of genera and families. Key genera and families are labelled on each graph.

Species diversity also varied markedly among sites (Fig. S2; species richness, *F*_3,45_ = 17.1, *P *<* *0.001; Fisher's alpha, *F*_3,45_ = 12.4, *P *<* *0.001; Shannon index, *F*_3,45_ = 6.6, *P *<* *0.001). Highest understorey species richness and diversity were found in forests in northern Peru and Manaus (Fig. S2). Patterns of species composition among the four sites were similar to variation reported for trees ≥10 cm diameter with some families (e.g. Myristicaceae, Moraceae, Violaceae, Arecaceae) particularly common in western Amazon forests, and others (e.g. Lecthyidaceae, Sapotaceae, Chrysobalanaceae) more characteristic of central Amazon forests and forests on the Guiana Shield (Fig. 2). The differences in composition among sites are more marked at the genus‐level, where a number of genera of strictly understorey trees make important contributions to defining the overall patterns: *Geonoma* (Arecaceae), *Piper* (Piperaceae) and *Calyptranthes* (Myrtaceae) are common in the understorey of western Amazon forests and *Casearia* (Salicaceae) and *Pausandra* (Euphorbiaceae) are common in the sites close to Manaus (Fig. 2). In contrast, no strictly understorey genera are particularly common in the forests at Nouragues (Fig. 2).

**Figure 3:**
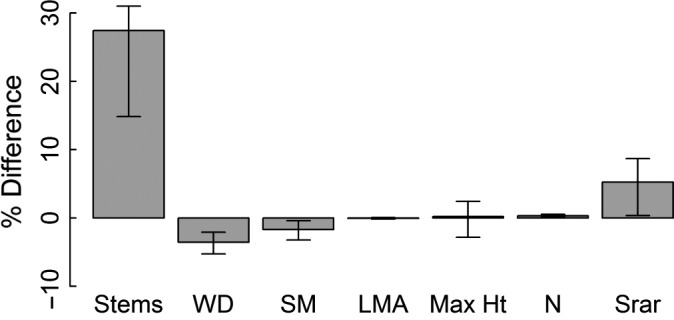
Mean and 95% confidence limits on percentage change in stem density, mean community‐level traits (WD – wood density, SM – Seed mass, LMA – leaf mass per unit area, Max Ht – maximum height and N – nitrogen concentration) and diversity (based on rarefied species richness) between disturbed and control subplots in four Amazonian forests.

The effect of disturbance on the composition of the understorey in each site was small and very similar compared to the substantial variation among the four forests (Figs 3 and S3). Across all four sites, there was a significant increase in stem number with 20.3 ± 7.6 more stems in the disturbed compared to the control subplots – overall, an increase of 20.9% (Fig. 3) – demonstrating that the disturbance events were followed by subsequent forest regrowth. In addition, the size of the gaps and timing of our sampling were appropriate for detecting pioneer species which very likely established as a result of the focal disturbance event. For example, stems of just five, strongly light‐demanding genera (*Carica, Cecropia, Cestrum, Vismia, Zanthoxylum*) were found in the disturbed subplots of almost half of the subplot pairs (23 of 49 pairs 47%); *Cecropia* was found in the disturbed subplot of 15 pairs. However, the only significant changes in the mean values of functional traits were decreases in community‐level wood density and seed mass between control and disturbed subplots and the magnitude of these changes was small (4 and 2%, respectively; Fig. 3). The only change that was significant within individual sites was a decrease in mean wood density in Nouragues (*t* = −3.55, *P *<* *0.01; Fig. S3). Mixed models confirmed that there were no significant differences among sites in their response to disturbance in any of the functional traits after accounting for the effect of the age and intensity of the disturbances (AIC values with/without site as a factor; wood density 161/159, likelihood ratio test, ns; max height 209/207, ns; seed mass 13.1/11.3, ns; leaf nitrogen 202/204, ns; specific leaf area 221/219, ns).

**Figure 4:**
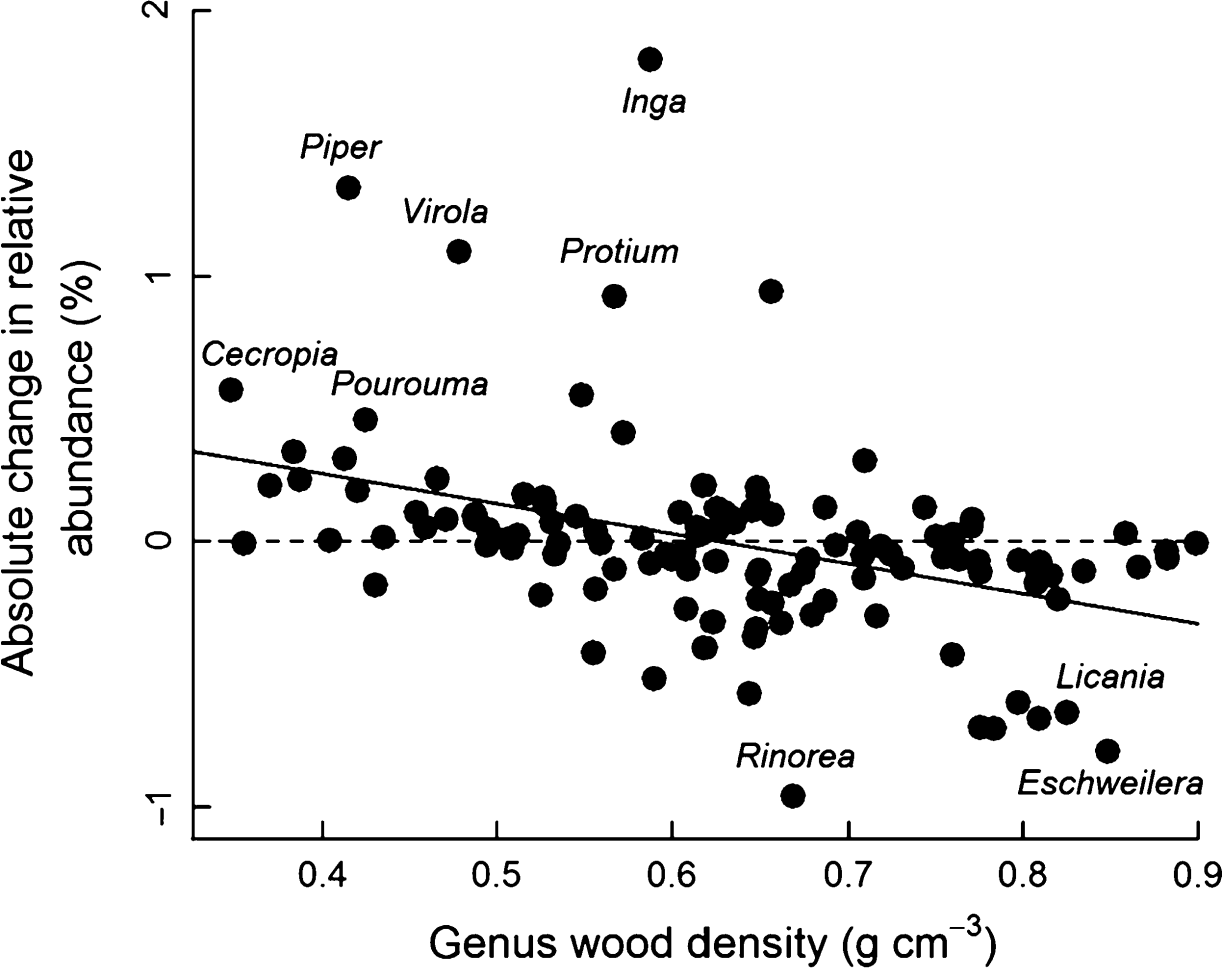
Relationship between the effect of disturbance on the relative abundance of 121 genera across four Amazonian forests and the mean wood density of those genera. Wood density data from Zanne *et al*. (2009). Linear regression line (Change in relative abundance = 0.7078–1.1312*Genus wood density) also shown.

Overall, there were no substantial changes in species composition between disturbed and control subplots within any forest: ordination of the composition of each site revealed very similar patterns between disturbed and control subplots (Fig. S4). However, there were changes in the relative abundance of some genera related to some of the functional traits. For example, mean genus wood density was significantly negatively correlated with the effect of disturbance: genera with low wood density such as *Cecropia*,* Pourouma*,* Inga* and *Piper* increased, whilst genera with high wood density such as *Eschweilera* and *Licania* declined in abundance (Fig. 4; *F*_1,119_ = 24.0, *P *<* *0.001).

**Figure 5:**
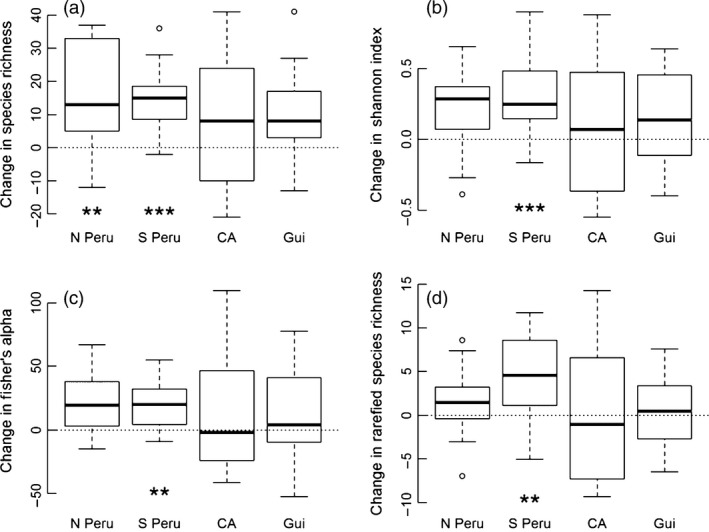
Difference in (a) species richness, (b) Shannon Index, (c) Fisher's alpha and (d) rarefied estimates of species richness between disturbed and control subplots in four Amazonian forests (north and south Peru, Central Amazonia (Manaus; CA) and the Guiana Shield (Nouragues; Gui). Positive values indicate higher values in the disturbed subplots. Significant differences for individual sites shown as *P < *0.01 (**) and *P *<* *0.001 (***).

Species richness was higher in disturbed subplots, similar to the changes in stem density (average change across all sites: 12.6 ± 4.7, Fig. 5). Overall, species diversity also increased marginally in disturbed subplots (Fig. 4), but this pattern was individually significant in only one site (S Peru, Fisher's alpha *t* = 4.11, *P *<* *0.005; Shannon index, *t* = 4.30, *P *<* *0.001, rarefied species richness *t* = 3.13, *P *<* *0.01; Fig. 5); the effect of disturbance on diversity did not vary significantly among sites (Fig. 5; AIC values with/without site as a factor; rarefied species richness 282/280, likelihood ratio test, ns; Fisher's alpha 479/477, ns; Shannon index, 58.1/57.1, ns). Treefall disturbance events also did not augment the variability of composition among disturbed, compared to undisturbed, patches of forest: the disturbed subplots did not show greater beta‐diversity – greater variation in composition – than the control subplots in any site (Fig. S4; permutation test for homogeneity of multivariate dispersion based on 999 permutations; N Peru, *P* = 0.98, S Peru, *P* = 0.36, Manaus, *P* = 0.74, Nouragues, *P* = 0.58).

## Discussion

Overall, these results show small and similar effects of treefall disturbance events on the functional composition and diversity of four contrasting sites in Amazonia.

The findings from the control plots show that previously reported large differences in the diversity, and species and functional composition of canopy trees in Amazonian forests (e.g. ter Steege *et al*. 2003, 2006) extend to the understorey. Additional families and genera contribute to these patterns (e.g. Piperaceae, *Pausandra*; Fig. 2), but overall the higher wood density, larger seed size and greater maximum height of species in forests on the Guiana Shield compared to forests in the western Amazon are apparent even when considering only the understorey of these sites (Fig. 1). Indeed, the low stem density, high maximum height and species composition of stems 2–10 cm diameter in Nouragues suggest that most understorey stems in this forest are juveniles of taller statured species, rather than a distinct understorey flora (Baker *et al*. 2009).

The overall differences in mean trait values between disturbed and control subplots (Fig. 3) indicate that species with lower wood density, and therefore presumably higher rates of diameter growth, and those with lighter seeds, and therefore greater dispersal ability, are favoured by disturbance (Figs 3 and 4). This finding is consistent with previous studies of disturbance events in tropical forests (Schnitzer & Carson 2001; Wright *et al*. 2003) and other systems (e.g. Cadotte 2007; Haddad *et al*. 2008). More surprising was that the magnitude of the response was similar across all sites – there was no difference in the effect of disturbance on any trait among the four sites (Fig. S3). In contrast, our hypothesis predicted that the resilience of different sites to disturbance would vary, depending on the underlying patterns of composition. In fact, evidence for this is very limited: Nouragues was the only site that individually showed a significant change in functional composition – a decline in wood density following disturbance (Fig. S3). This specific pattern is consistent with our prediction, but overall, in contrast to the large differences among these sites, there is little variation in how the functional composition of these forests responds to disturbance (Fig. S3).

This study also suggests that the impact of treefall disturbance events on alpha‐diversity is significant but small in Amazonian forests (Fig. 3). The disturbed plots showed similar, small increases in diversity in each site, irrespective of the measure used to quantify diversity (Fig. 5). This pattern is related to the small changes in functional composition of these stands following disturbance. Although disturbance did favour the growth of some taxa with specific functional traits such as low wood density (Fig. 4), the fact that few species in tropical forests are strictly dependent on gap formation for regeneration (e.g. Wright *et al*. 2003) is likely to have precluded strong effects of disturbance on diversity. In general, the proportion of species in any community at different points along the competition/colonization trade‐off is likely to be the crucial factor for determining how disturbance influences diversity (Cadotte 2007; Haddad *et al*. 2008). The experimental and modelling studies that have been used to demonstrate the importance of functional composition for predicting the effect of disturbance have used a wide range of comparatively extreme treatments to demonstrate these effects (Cadotte 2007; Haddad *et al*. 2008). In contrast, based on this study, in tropical forests, where few species occur at the extremes of this trade‐off and many species are intermediate, strong positive effects of treefall disturbance events on diversity appear unlikely.

Although the effects of disturbance on alpha‐diversity were generally small, in southern Peru, there was a significant, positive impact of disturbance on diversity (Fig. 5). In this site, the increase in diversity is related to the sensitivity of the most common shade‐tolerant species to disturbance: the most abundant species, *Rinorea viridifolia* (Violaceae), declined markedly in relative abundance, from 7.7 to 3.3%, between control and disturbed subplots. The architectural strategy of this species may be the most important trait for defining how this species responds to disturbance. *Rinorea viridifolia* has plagiotropic branching (Terborgh & Matthews 1999) that is easily overtopped by other species in forest patches that are vigorously regenerating. Observations of *R. viridifolia* suggest that this species can survive partial uprooting and stem breakage due to treefall events, but its horizontal branching means that it is easily outcompeted and dies in the deep shade of a regenerating gap. The characteristics of this species mean that, in this case, disturbance acts to favour the rarer species in the community – by reducing the abundance of the most common species. This is an example of a ‘stabilizing’ mechanism, distinct from competition–colonization trade‐offs, that allows disturbance to cause stable, long‐term, coexistence of species (Fox 2013). This process may operate over a substantial area of Amazonia as *R. viridifolia* is one of the abundant ‘hyperdominant’ tree species of southwest Amazon forests (ter Steege *et al*. 2013). The impact of this species on the disturbance ecology of these forests demonstrates how understanding the behaviour of the common tree species of Amazonia can help to elucidate how these ecosystems function (ter Steege *et al*. 2013; Fauset *et al*. 2015). However, it is worth emphasizing that the contribution of this mechanism to maintaining diversity even in this region is still small (Fig. 5) and that it does not operate in other regions, where common species are not preferentially killed by disturbance events or subsequent forest regeneration.

Our study also suggests that the effect of disturbance on beta‐diversity, as well as alpha‐diversity, is small in these sites. The effect of disturbance on beta‐diversity has been far less studied than the effect on diversity at the scale of individual patches, but can also be important because dispersal limitation can strongly influence diversity/disturbance relationships (ter Steege, Welch & Zagt 2002; Cadotte 2007). More specifically, if species are unable to reach all suitable patches in the landscape, species turnover among regenerating patches of forests may be higher than within undisturbed forest (Hubbell *et al*. 1999; Köhler & Huth 2007). However, this study found no difference in beta‐diversity between disturbed and control subplots in any site (Fig. S4). This pattern is probably because only a small proportion of the stems in disturbed subplots resulted from colonization following the focal disturbance event, and therefore, the potential impact of dispersal limitation on diversity is low.

Overall, our study suggests that small‐scale, treefall disturbances make only a small contribution to maintaining patterns of diversity in these sites by allowing the coexistence of species with different traits via the competition/colonization trade‐off, ‘stabilizing’ mechanisms or dispersal limitation (Figs 5 and S4). These results are consistent with the patterns reported by Hubbell *et al*. (1999) for a single site in Panama, and from a broader scale analysis of Ghanaian tropical forest showing that correlates of disturbance intensity explain only a small proportion of alpha‐diversity across an extensive forest plot network (Bongers *et al*. 2009). However, these findings contrast with the significant effects of disturbance that have been reported following logging in French Guiana (Molino & Sabatier 2001). One reason for this difference is the range of intensities of disturbance events that have been studied; studies based on logged forest include larger and more extreme disturbances than we included here (Sheil & Burslem 2003). In these settings, small patches may become dominated by light‐demanding, pioneer species which depress overall diversity and substantially alter functional composition; this pattern contrasts to our study where many individuals survived the disturbance events in each patch. Large and severe disturbance events due to blowdowns can cause similar large changes in diversity and composition in Amazonian forests (Chambers *et al*. 2009), which may mean that comparisons at certain locations and scales may reveal stronger effects of disturbance on forest composition. However, the rarity of large disturbance events in Amazonian forests may limit their role as a mechanism maintaining the broad variation in species composition and diversity across this region: one‐hectare disturbances have a return time of 10 000 years (Espírito‐Santo *et al*. 2014) compared to average estimated generation times of Amazonian trees of tens to hundreds of years (Baker *et al*. 2014).

Our findings here also contrast with previously reported correlations between diversity and stand‐level, stem turnover rates in Amazonia forests that were used to argue for a positive effect of disturbance on species richness (Phillips *et al*. 1994). The results of our study suggest that this correlation does not reflect the processes that maintain patterns of diversity today. In contrast, this relationship may be the outcome of historical processes that have generated these patterns of diversity. Species‐rich clades of Amazonian trees are characterized by relatively short life spans and make a particularly important contribution to the high diversity of western Amazon forests (Baker *et al*. 2014). The high abundance of these lineages in western Amazonia may have been favoured by the comparatively rich soils with poor physical properties that result in these forests having high stand‐level mortality rates (Quesada *et al*. 2012; Baker *et al*. 2014). The correlation reported by Phillips *et al*. (1994) may therefore result from high, long‐term, stand‐level mortality rates promoting diversification over evolutionary time scales.

In summary, this study has a range of implications for understanding the role of small‐scale treefall disturbance events for determining the diversity and composition of moist tropical forests, both today and in the future. Firstly, this study suggests that these disturbances play only a small role as a mechanism that maintains current diversity patterns at small spatial scales within and among Amazonian forests. However, correlations between measures of disturbance and diversity may still be found at larger scales (e.g. Phillips *et al*. 1994), where the measures of disturbance may reflect the legacy of historical processes (Baker *et al*. 2014) or the effect of other underlying coexistence mechanisms (Huston 2014). If these correlations are indeed indirect relationships, then they may not be found for tropical forests in different settings on other continents (e.g. Bongers *et al*. 2009). In contrast to their effect on diversity, treefall disturbance events may have a stronger role in determining the functional composition of Amazonian forests by altering the abundance of a suite of disturbance‐adapted taxa (e.g. Fig. 4). This result is consistent with theoretical models which demonstrate how variation in long‐term average mortality rates can alter the dominant species in an ecosystem (Fox 2013), and empirical correlations between stand mortality rates and mean wood density across Amazonian forests (Baker *et al*. 2004; Quesada *et al*. 2012). As a result, the functional composition, rather than diversity, of intact Amazonian forests may be most likely to alter as treefall disturbance regimes change in the future.

## Data accessibility

Data on the diversity, structure, floristics and functional composition of the control and disturbed subplots used in this study are available at ForestPlots.net (Baker *et al*. 2015).

## Supporting information


**Table S1.** Stem and species number, mean wood density and diversity for each subplot pair.
**Figure S1.** Mortality rates and 95% confidence limits calculated on a basal area basis for the 48 pairs of control (undisturbed) and disturbed 20 × 20 m subplots used in this study.
**Figure S2.** Box plots of variation in (a) species richness, (b) Fisher's alpha and (c) Shannon index for stems 2–10 cm diameter in four Amazonian forests.
**Figure S3.** Box plots of differences in (a) stem density and mean values of (b) wood density, (c) leaf mass per unit area, (d) seed mass, (e) maximum height, (f) nitrogen concentration between disturbed and control subplots for stems 2–10 cm diameter in forests in four Amazonian forests.
**Figure S4.** Variation in the species composition between disturbed and control subplots for four Amazonian forests
