# Supplementary material for: Consistent, small effects of treefall disturbances on the composition and diversity of four Amazonian forests
Source: J Ecol. 2016 Jan 17;104(2):497–506. doi: 10.1111/1365-2745.12529 (PMC4991291; doi:10.1111/1365-2745.12529)
Supplement: Supplementary file 1 — Table S1. Stem and species number, mean wood density and diversity for each subplot pair. Figure S1. Mortality rates and 95% confidence limits calculated on a basal area basis for the 48 pairs of control (undisturbed) and disturbed 20 × 20 m subplots used in this study. Figure S2. Box plots of variation in (a) species richness, (b) Fisher's alpha and (c) Shannon index for stems 2–10 cm diameter in four Amazonian forests. Figure S3. Box plots of differences in (a) stem density and mean values of (b) wood density, (c) leaf mass per unit area, (d) seed mass, (e) maximum height, (f) nitrogen concentration between disturbed and control subplots for stems 2–10 cm diameter in forests in four Amazonian forests. Figure S4. Variation in the species composition between disturbed and control subplots for four Amazonian forests [file JEC-104-497-s001.docx]

**Supporting information**

**Table S1.** Stem and species number, mean wood density and diversity for each subplot pair. Estimates of diversity based on rarefaction using the lowest number of stems from any subplot in each site. Some plots contain more than one disturbed and control subplot pair; some subplot pairs are split between two nearby long-term monitoring plots. SP, subplot number; S, stem number; Spp, species richness; MWD, mean wood density; D, diversity. Subscripts refer to control (c) or disturbed (d) subplots respectively.

|  |  | Control subplot | | | | | | | | Disturbed subplot | | | | | | | |
| --- | --- | --- | --- | --- | --- | --- | --- | --- | --- | --- | --- | --- | --- | --- | --- | --- | --- |
| Site | Pair | Plot | Lat. | Long. | SP_c_ | S_c_ | Spp_c_ | MWD_c_ | D_c_ | Plot | Lat. | Long. | SP_d_ | S_d_ | Spp_d_ | MWD_d_ | D_d_ |
| N Peru | 7 | ALP-11 | -3.95 | -73.43 | 6 | 88 | 48 | 0.59 | 37 | ALP-11 | -3.95 | -73.43 | 1 | 125 | 48 | 0.55 | 30 |
|  | 8 | ALP-12 | -3.95 | -73.44 | 22 | 123 | 85 | 0.65 | 48 | ALP-12 | -3.95 | -73.44 | 19 | 189 | 120 | 0.60 | 50 |
|  | 11 | YAN-01 | -3.44 | -72.85 | 9 | 62 | 41 | 0.64 | 41 | YAN-01 | -3.44 | -72.85 | 7 | 78 | 50 | 0.58 | 42 |
|  | 12 | SUC-05 | -3.25 | -72.90 | 7 | 95 | 52 | 0.59 | 38 | SUC-05 | -3.25 | -72.90 | 1 | 99 | 65 | 0.60 | 46 |
|  | 13 | SUC-05 | -3.25 | -72.90 | 19 | 120 | 66 | 0.62 | 41 | SUC-05 | -3.25 | -72.90 | 4 | 100 | 54 | 0.55 | 38 |
|  | 14 | SUC-04 | -3.25 | -72.90 | 17 | 87 | 57 | 0.57 | 43 | SUC-04 | -3.25 | -72.90 | 13 | 120 | 76 | 0.59 | 45 |
|  | 15 | SUC-04 | -3.25 | -72.90 | 16 | 144 | 80 | 0.60 | 42 | SUC-04 | -3.25 | -72.90 | 6 | 130 | 91 | 0.59 | 49 |
|  | 16 | SUC-02 | -3.26 | -72.90 | 13 | 119 | 78 | 0.59 | 46 | SUC-02 | -3.26 | -72.90 | 24 | 168 | 110 | 0.58 | 49 |
|  | 17 | SUC-02 | -3.26 | -72.90 | 21 | 93 | 62 | 0.61 | 46 | SUC-02 | -3.26 | -72.90 | 19 | 135 | 96 | 0.59 | 50 |
|  | 18 | ALP-30 | -3.95 | -73.43 | 13 | 162 | 54 | 0.64 | 30 | ALP-30 | -3.95 | -73.43 | 22 | 176 | 55 | 0.63 | 29 |
|  | 19 | YAN-02 | -3.43 | -72.84 | 6 | 90 | 62 | 0.62 | 47 | YAN-02 | -3.43 | -72.84 | 9 | 157 | 99 | 0.63 | 47 |
| S Peru | 1 | TAM-05 | -12.83 | -69.27 | 15 | 113 | 43 | 0.62 | 31 | TAM-05 | -12.83 | -69.27 | 24 | 89 | 50 | 0.62 | 39 |
|  | 2 | TAM-01 | -12.84 | -69.28 | 11 | 98 | 57 | 0.63 | 42 | TAM-01 | -12.84 | -69.28 | 2 | 118 | 73 | 0.59 | 45 |
|  | 3 | TAM-02 | -12.83 | -69.28 | 18 | 99 | 40 | 0.64 | 31 | TAM-02 | -12.83 | -69.28 | 6 | 127 | 66 | 0.62 | 43 |

Supporting Table 1. (cont.)

|  |  | Control subplot | | | | | | | | Disturbed subplot | | | | | | | |
| --- | --- | --- | --- | --- | --- | --- | --- | --- | --- | --- | --- | --- | --- | --- | --- | --- | --- |
| Site | Pair | Plot | Lat. | Long. | SP_c_ | S_c_ | Spp_c_ | WD_c_ | D_c_ | Plot | Lat. | Long. | SP_d_ | S_d_ | Spp_d_ | WD_d_ | D_d_ |
| S Peru | 4 | TAM-01 | -12.84 | -69.28 | 25 | 83 | 47 | 0.61 | 38 | TAM-01 | -12.84 | -69.28 | 5 | 86 | 62 | 0.64 | 48 |
|  | 5 | TAM-02 | -12.83 | -69.28 | 1 | 94 | 47 | 0.62 | 36 | TAM-02 | -12.83 | -69.28 | 4 | 99 | 56 | 0.64 | 40 |
|  | 6 | CUZ-01 | -12.50 | -68.97 | 19 | 88 | 44 | 0.58 | 35 | CUZ-01 | -12.50 | -68.97 | 21 | 105 | 42 | 0.54 | 30 |
|  | 9 | CUZ-02 | -12.50 | -68.97 | 9 | 68 | 31 | 0.61 | 29 | CUZ-02 | -12.50 | -68.97 | 15 | 141 | 46 | 0.50 | 28 |
|  | 10 | CUZ-04 | -12.50 | -68.96 | 5 | 114 | 56 | 0.60 | 39 | CUZ-04 | -12.50 | -68.96 | 8 | 97 | 59 | 0.64 | 43 |
|  | 41 | MNU-01 | -11.87 | -71.35 | 2/2,3/2 | 75 | 33 | 0.62 | 29 | MNU-01 | -11.87 | -71.35 | 1/5,2/5 | 140 | 69 | 0.60 | 38 |
|  | 42 | MNU-05 | -11.87 | -71.35 | 8 | 96 | 56 | 0.63 | 42 | MNU-05 | -11.87 | -71.35 | 2 | 162 | 71 | 0.53 | 37 |
|  | 43 | MNU-05 | -11.87 | -71.35 | 12 | 80 | 44 | 0.62 | 37 | MNU-05 | -11.87 | -71.35 | 16 | 123 | 72 | 0.60 | 46 |
|  | 44 | MNU-05 | -11.87 | -71.35 | 21 | 76 | 44 | 0.60 | 39 | MNU-05 | -11.87 | -71.35 | 18 | 97 | 65 | 0.63 | 47 |
|  | 45 | MNU-06 | -11.87 | -71.35 | 11 | 76 | 47 | 0.60 | 41 | MNU-06 | -11.87 | -71.35 | 30 | 83 | 60 | 0.54 | 48 |
|  | 46 | MNU-06 | -11.87 | -71.35 | 16 | 65 | 37 | 0.55 | 36 | MNU-06 | -11.87 | -71.35 | 29 | 105 | 53 | 0.56 | 38 |
|  | 47 | MNU-06 | -11.87 | -71.35 | 14 | 62 | 39 | 0.59 | 39 | MNU-06 | -11.87 | -71.35 | 33 | 81 | 47 | 0.57 | 39 |
| Manaus | 28 | BDF-06 | -2.41 | -59.85 | 191 | 119 | 79 | 0.67 | 54 | BDF-06 | -2.41 | -59.85 | 255 | 178 | 93 | 0.61 | 45 |
|  | 29 | BDF-06 | -2.41 | -59.85 | 120 | 72 | 50 | 0.69 | 50 | BDF-06 | -2.41 | -59.85 | 178 | 112 | 76 | 0.66 | 53 |
|  | 30 | BDF-06 | -2.41 | -59.85 | 238 | 110 | 62 | 0.67 | 44 | BDF-06 | -2.41 | -59.85 | 209 | 133 | 86 | 0.65 | 52 |
|  | 31 | BDF-07 | -2.40 | -59.90 | 18 | 119 | 62 | 0.66 | 43 | BDF-07 | -2.40 | -59.90 | 4 | 144 | 103 | 0.64 | 57 |
|  | 32 | BDF-07 | -2.40 | -59.90 | 12 | 93 | 61 | 0.68 | 50 | BDF-07 | -2.40 | -59.90 | 20 | 113 | 69 | 0.66 | 47 |
|  | 33 | BDF-12 | -2.39 | -59.85 | 190 | 117 | 77 | 0.65 | 51 | BDF-12 | -2.39 | -59.85 | 180 | 77 | 64 | 0.68 | 60 |
|  | 34 | BDF-12 | -2.39 | -59.85 | 188 | 82 | 61 | 0.66 | 55 | BDF-12 | -2.39 | -59.85 | 195 | 119 | 82 | 0.62 | 56 |

Supporting Table 1. (cont.)

|  |  | Control subplot | | | | | | | | Disturbed subplot | | | | | | | |
| --- | --- | --- | --- | --- | --- | --- | --- | --- | --- | --- | --- | --- | --- | --- | --- | --- | --- |
| Site | Pair | Plot | Lat. | Long. | SP_c_ | S_c_ | Spp_c_ | WD_c_ | D_c_ | Plot | Lat. | Long. | SP_d_ | S_d_ | Spp_d_ | WD_d_ | D_d_ |
| Manaus | 35 | BDF-12 | -2.39 | -59.85 | 185 | 103 | 72 | 0.64 | 60 | BDF-12 | -2.39 | -59.85 | 197 | 153 | 111 | 0.63 | 60 |
|  | 36 | BDF-13 | -2.40 | -59.91 | 20 | 124 | 81 | 0.70 | 44 | BDF-13 | -2.40 | -59.91 | 13 | 149 | 78 | 0.67 | 44 |
|  | 37 | BDF-13 | -2.40 | -59.91 | 129 | 128 | 75 | 0.63 | 45 | BDF-13 | -2.40 | -59.91 | 139 | 133 | 71 | 0.67 | 45 |
|  | 38 | BDF-13 | -2.40 | -59.91 | 198 | 121 | 70 | 0.67 | 38 | BDF-13 | -2.40 | -59.91 | 166 | 135 | 60 | 0.64 | 38 |
|  | 39 | BDF-13 | -2.40 | -59.91 | 173 | 127 | 70 | 0.66 | 41 | BDF-13 | -2.40 | -59.91 | 160 | 110 | 57 | 0.65 | 41 |
|  | 40 | BDF-13 | -2.40 | -59.91 | 90 | 145 | 91 | 0.69 | 45 | BDF-13 | -2.40 | -59.91 | 156 | 128 | 70 | 0.67 | 45 |
| Nouragues | 20 | NOR-11 | 4.09 | -52.67 | 25 | 93 | 60 | 0.69 | 33 | NOR-10 | 4.09 | -52.67 | 24 | 113 | 65 | 0.62 | 31 |
|  | 21 | NOR-11 | 4.09 | -52.67 | 23 | 99 | 49 | 0.70 | 29 | NOR-10 | 4.09 | -52.67 | 3 | 91 | 66 | 0.66 | 36 |
|  | 22 | NOR-12 | 4.09 | -52.67 | 4 | 63 | 37 | 0.68 | 29 | NOR-10 | 4.09 | -52.67 | 17 | 85 | 48 | 0.64 | 30 |
|  | 23 | NOR-12 | 4.09 | -52.67 | 8 | 60 | 38 | 0.65 | 29 | NOR-11 | 4.08 | -52.67 | 4 | 67 | 41 | 0.69 | 30 |
|  | 24 | NOR-20 | 4.08 | -52.67 | 11 | 58 | 35 | 0.75 | 29 | NOR-20 | 4.08 | -52.67 | 21 | 70 | 50 | 0.66 | 35 |
|  | 25 | NOR-21 | 4.08 | -52.67 | 15 | 56 | 42 | 0.70 | 34 | NOR-21 | 4.08 | -52.67 | 17 | 63 | 45 | 0.67 | 34 |
|  | 26 | NOR-20 | 4.08 | -52.67 | 9 | 43 | 32 | 0.70 | 32 | NOR-21 | 4.08 | -52.67 | 7 | 89 | 59 | 0.65 | 34 |
|  | 27 | NOR-21 | 4.08 | -52.67 | 16 | 79 | 57 | 0.65 | 35 | NOR-21 | 4.08 | -52.67 | 24 | 81 | 44 | 0.65 | 29 |
|  | 48 | NOR-21 | 4.08 | -52.67 | 19 | 103 | 64 | 0.73 | 32 | NOR-21 | 4.08 | -52.67 | 12 | 121 | 62 | 0.68 | 27 |
|  | 49 | NOR-21 | 4.08 | -52.67 | 25 | 58 | 43 | 0.73 | 34 | NOR-21 | 4.08 | -52.67 | 16 | 112 | 84 | 0.67 | 37 |

**Figure S1.** Mortality rates and 95 % confidence limits calculated on a basal area basis for the 48 pairs of control (undisturbed) and disturbed 20 x 20 m subplots used in this study, based on inventory data for trees ≥10 cm diameter.

**
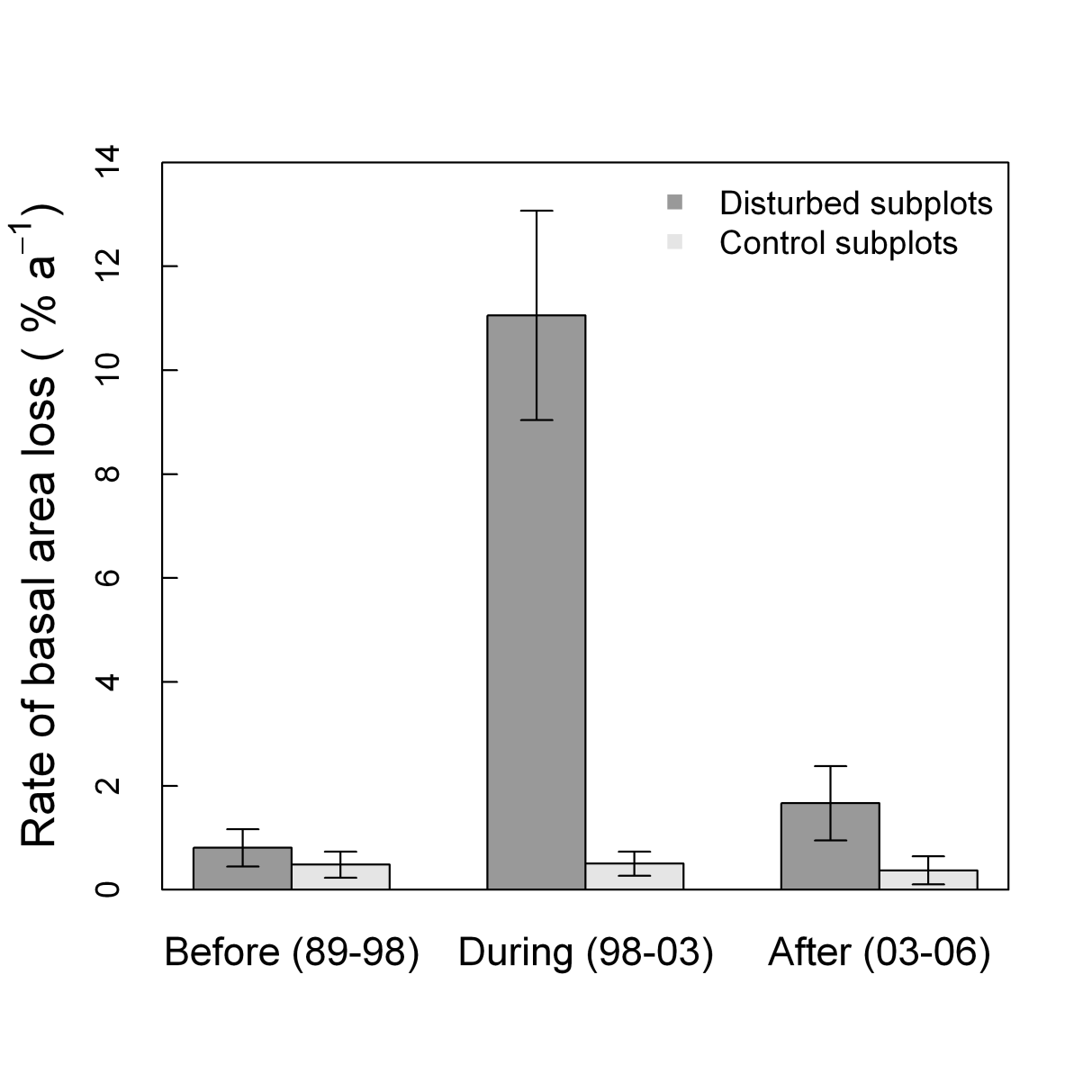
**


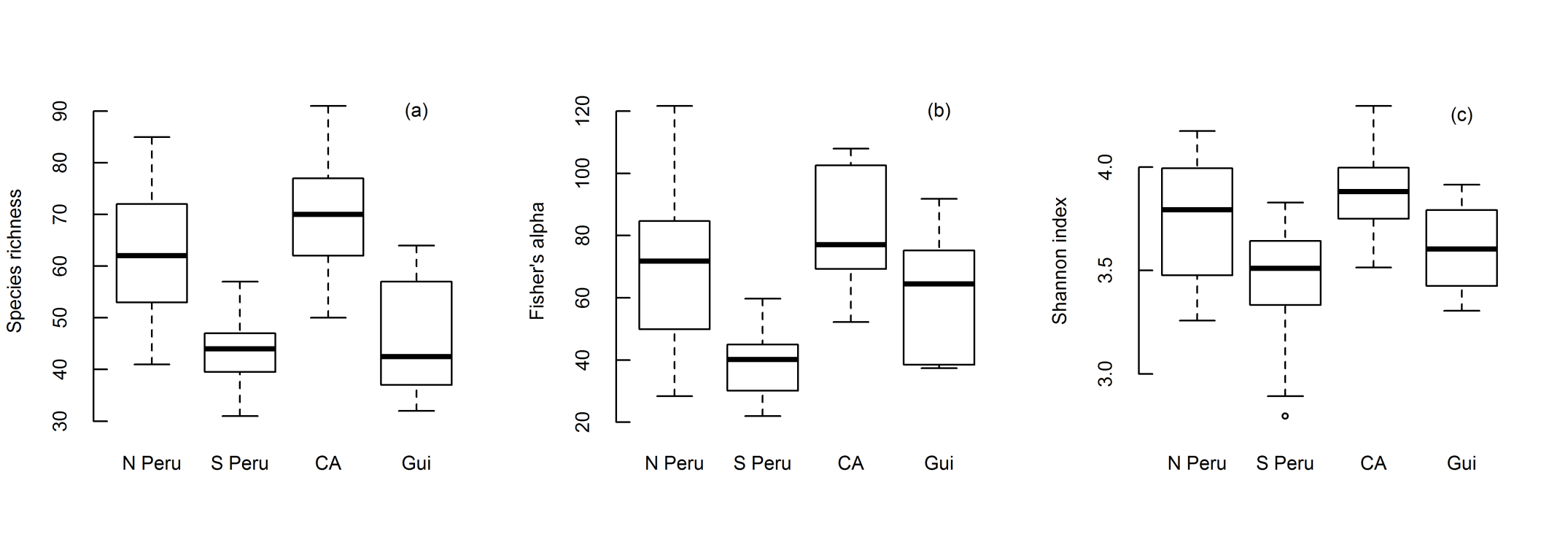
**Figure S2**. Boxplots of variation in (a) species richness, (b) Fisher’s alpha and (c) Shannon index for stems 2-10 cm diameter in four Amazonian forests (north and south Peru, Central Amazonia (Manaus; CA) and the Guiana Shield (Nouragues; Gui). Differences among sites are significant (*P* < 0.001) for each variable.


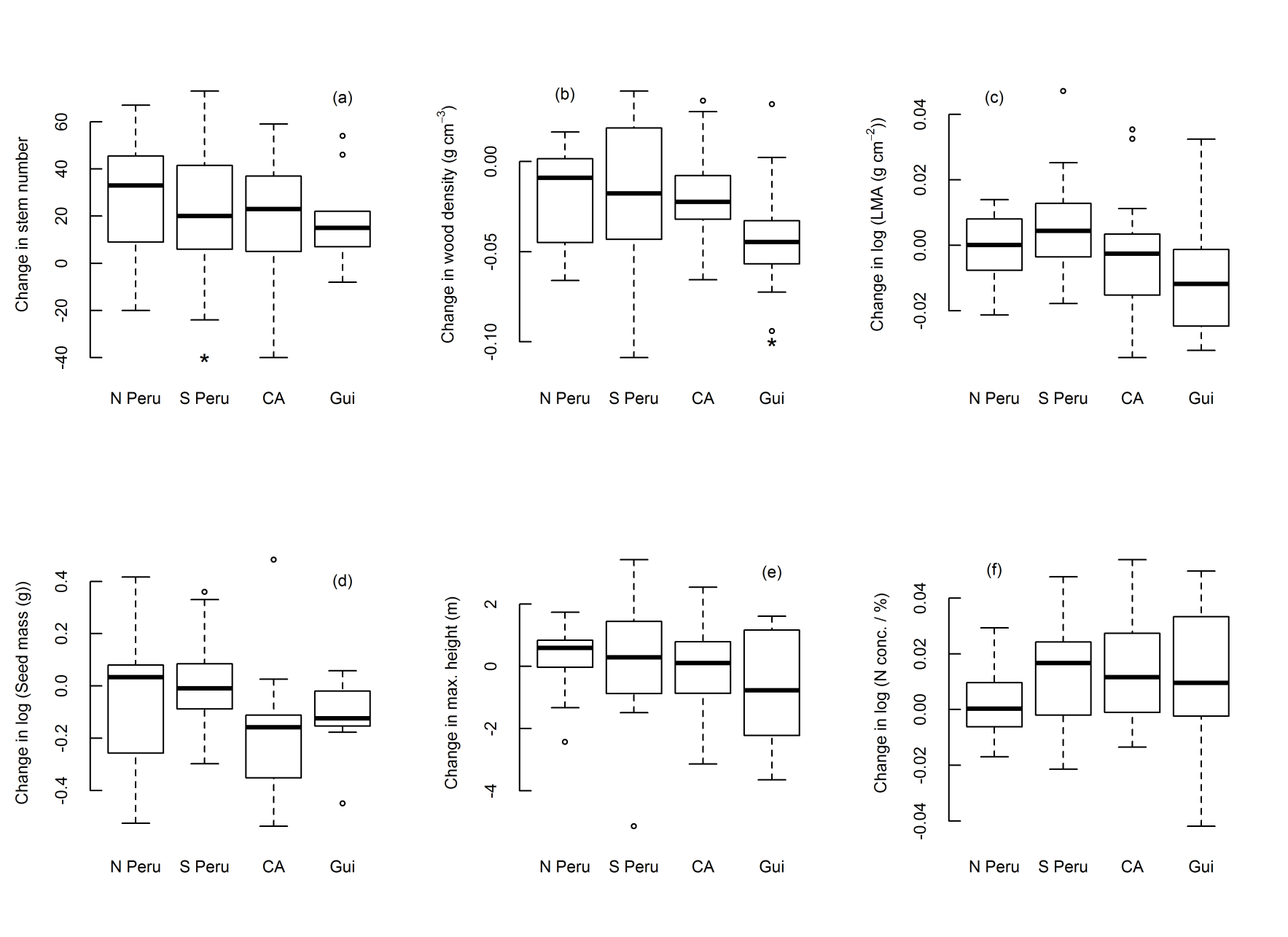
**Figure S3.** Boxplots of differences in (a) stem density and mean values of (b) wood density, (c) leaf mass per unit area, (d) seed mass, (e) maximum height, (f) nitrogen concentration between disturbed and control subplots for stems 2-10 cm diameter in forests in four Amazonian forests (north and south Peru, Central Amazonia (Manaus; CA) and the Guiana Shield (Nouragues; Gui). Positive values indicate higher values in the disturbed subplots. Significant changes in individual sites shown as *P* < 0.01 (*).

**Figure S4.** Variation in the species composition between disturbed (circles) and control (triangles) subplots for four Amazonian forests (a: north Peru, b: south Peru, c: Manaus, central Amazonia, d: Nouragues, Guiana Shield) based on a principal coordinates analysis of composition using the Bray Curtis measure of similarity. Within each ordination, the polygons connect either disturbed or control subplots to demonstrate their occupancy of multivariate space. The centroid (red) is also shown for both groups of subplots.


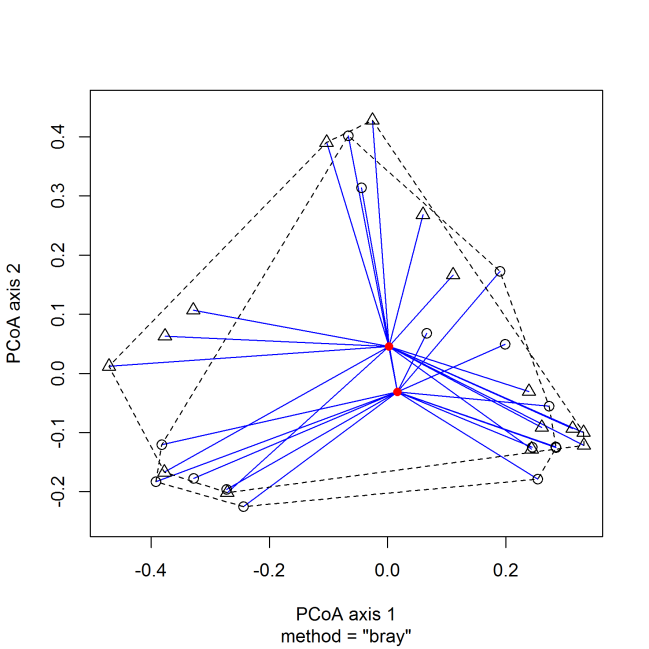

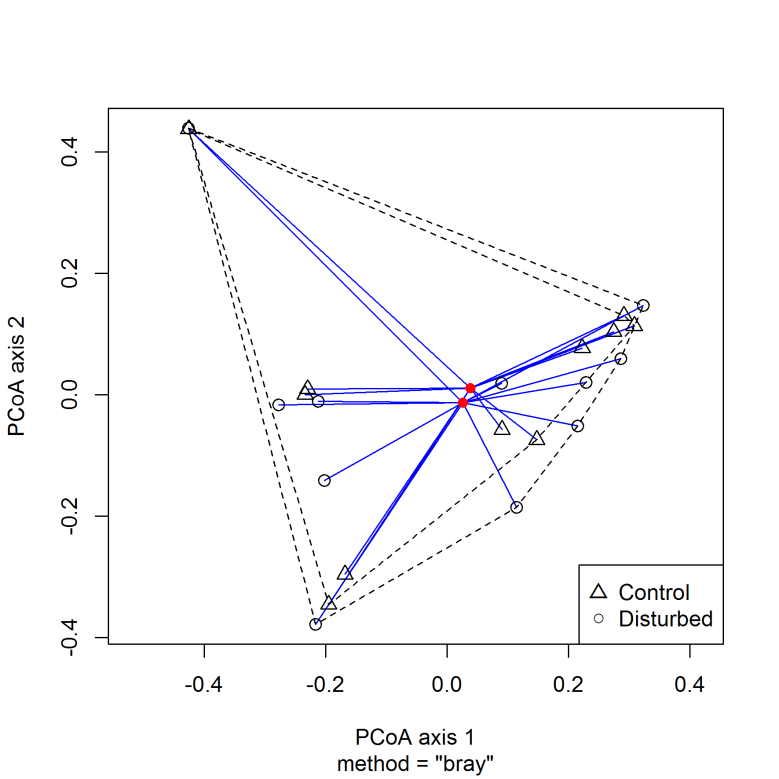

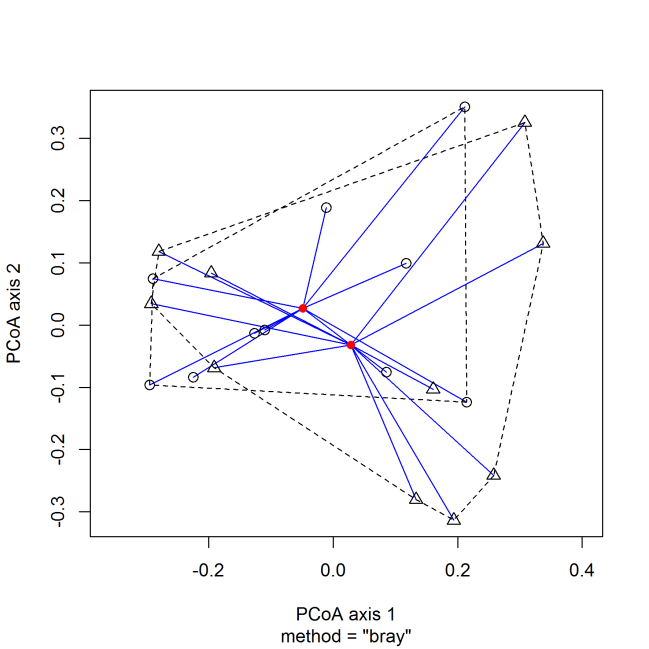

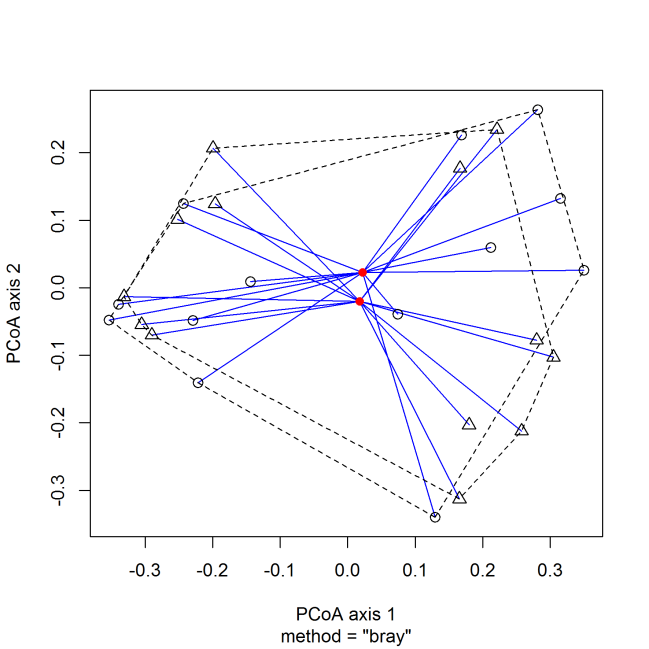


a)

b

c

d
